# NKG2D ligand RAE1ε induces generation and enhances the inhibitor function of myeloid‐derived suppressor cells in mice

**DOI:** 10.1111/jcmm.13124

**Published:** 2017-03-09

**Authors:** Li Qian, Yang Liu, Shaoqing Wang, Weijuan Gong, Xiaoqin Jia, Lu Liu, Feng Ye, Jingjuan Ding, Yuwei Xu, Yi Fu, Fang Tian

**Affiliations:** ^1^ Department of Immunology School of Medicine Yangzhou University Yangzhou China; ^2^ Translational Medicine Research Institute of Yangzhou University Yangzhou China; ^3^ Jiangsu Key Laboratory of Zoonosis/Jiangsu Co‐innovation Center for Prevention and Control of Important Animal Infectious Diseases and Zoonoses Yangzhou China; ^4^ Jiangsu Key Laboratory of Integrated Traditional Chinese and Western Medicine for Prevention and Treatment of Senile Diseases Yangzhou China

**Keywords:** NKG2D, myeloid‐derived suppressor cells, IL‐10, immunosuppression

## Abstract

Expression of surface NKG2D ligands on tumour cells, which activates nature killer (NK) cells and CD8^+^ T cells, is crucial in antitumour immunity. Some types of tumours have evolved mechanisms to suppress NKG2D‐mediated immune cell activation, such as tumour‐derived soluble NKG2D ligands or sustained NKG2D ligands produced by tumours down‐regulate the expression of NKG2D on NK cells and CD8^+^ T cells. Here, we report that surface NKG2D ligand RAE1ε on tumour cells induces CD11b^+^Gr‐1^+^ myeloid‐derived suppressor cell (MDSC) *via* NKG2D *in vitro* and *in vivo*. MDSCs induced by RAE1ε display a robust induction of IL‐10 and arginase, and these MDSCs show greater suppressive activity by inhibiting antigen‐non‐specific CD8^+^ T‐cell proliferation. Consistently, upon adoptive transfer, MDSCs induced by RAE1ε significantly promote CT26 tumour growth in IL‐10‐ and arginase‐dependent manners. RAE1ε moves cytokine balance towards Th2 but not Th1 *in vivo*. Furthermore, RAE1ε enhances inhibitory function of CT26‐derived MDSCs and promotes IL‐4 rather than IFN‐γ production from CT26‐derived MDSCs through NKG2D *in vitro*. Our study has demonstrated a novel mechanism for NKG2D ligand^+^ tumour cells escaping from immunosurveillance by facilitating the proliferation and the inhibitory function of MDSCs.

## Introduction

Ligands for NKG2D, including RAE1 (RAE1α‐RAE1ε), Mult1, H60, human MICA and MICB, are expressed on most of the tumour cell lines and in tumour tissues 1. Ligand activation of NKG2D is critical for antitumour immune response through activating NK cells and CD8^+^ T cells 2. However, some types of tumours have evolved mechanisms to evade from NKG2D‐mediated immunosurveillance. Several potential mechanisms have been proposed, including tumour‐derived soluble NKG2D ligands down‐regulating NKG2D expression on cytolytic cells, and sustained NKG2D ligands expression by tumours eliciting NKG2D down‐regulation which resulting in impairment of NKG2D‐dependent cell activation 3, 4. Nevertheless, the underlying mechanisms of NKG2D ligands‐mediated immune escape have not been well defined.

Myeloid‐derived suppressor cells (MDSCs) play important roles in tumour immune escape due to their suppressive function in modulating immune responses 5, 6. It is well accepted that MDSCs are heterogeneous populations, which include myeloid progenitors and immature myeloid cells that express Gr‐1^+^ and CD11b^+^ myeloid markers in mice 7, 8, 9. They exhibit a remarkable ability to depress T‐cell proliferative responses induced by anti‐CD3/anti‐CD28 antibodies, alloantigens or lectins 9, 10. MDSCs take various strategies to depress T‐cell function, including nitric oxide production, arginase expression, IL‐10 or TGF‐β secretion, regulatory T‐cell induction and prostaglandin E_2_ up‐regulation 5, 11, 12. Numerous studies have ascribed that tumour cells could produce some cytokines, such as GM‐CSF, and IL‐4, to support MDSCs generation, expansion and activity 11, 12. However, very limited information is available regarding tumour surface molecules inducing MDSCs accumulation and activation.

In this report, we have shown that NKG2D ligand RAE1ε expressed on tumour cells facilitates expansion and activation of immunosuppressive MDSCs *in vitro* and *in vivo*, thereby providing a novel mechanism by which NKG2D ligands inhibit immunosurveillance and lead to tumour progression.

## Materials and methods

### Mice

BALB/c mice (female, 6–8 weeks) were obtained from the Experimental Animal Center of Yangzhou University (Yangzhou, China). All animal procedures have been approved by the Animal Care and Use Committee of Yangzhou University (Yangzhou, China).

### BaF3 cells and RAE1ε stable‐overexpression

The murine IL‐3‐dependent pro‐B cell line BaF3 cells were cultured in RPMI 1640 medium with 10% foetal bovine serum and 1 ng/ml recombinant murine IL‐3 as described 13. RAE1ε‐overexpressed BaF3 cells were constructed as described in our previous study 14. Briefly, BaF3 cells were transfected with pVITRO2‐mcs/RAE1ε plasmid (RAE1ε transfectant) or pVITRO2‐mcs plasmid (mock‐transfectant) using jetPEI™ DNA transfection reagent (Polyplus‐transfection Inc) to get the BaF3‐RAE1ε and BaF3‐mock cells, respectively. After 48 hrs, RAE1ε‐ and mock‐transfected cells were cultured and selected by hygromycin B for 2 weeks. Stable BaF3‐RAE1ε cells were sorted for high expression of RAE1ε by flow cytometry.

### Subcutaneous tumour injection

BALB/c mice were injected subcutaneously with 4 × 10^6^ BaF3‐mock or BaF3‐RAE1ε cells resuspended in PBS at day 0. In some groups, mice were injected with neutralizing anti‐NKG2D antibody (250 μg/mouse; R&D Systems, Minneapolis, MN, USA) or isotype‐matched control mAb (250 μg/mouse) through tail vein every 3 days. As indicated in some experiments, BALB/c mice were injected subcutaneously with 2 × 10^6^ CT26 cells resuspended in PBS. At day 28 after injection, mice were killed for sorting MDSCs.

### Cell purification

MDSCs were isolated from erythrocyte‐depleted splenocytes using antimouse CD11b beads (Miltenyi Biotech, Bergisch Gladbach, Germany) following the manufacturer's instructions. In some experiments, MDSCs were sorted by FACSAria (BD Biosciences). The purity of MDSCs (CD11b^+^Gr‐1^+^) was typically >90%. Splenic CD8^+^ T cells were enriched using anti‐CD8 beads (Miltenyi Biotech). The purity of CD8^+^ cells was typically >95%.

### Flow cytometry

The fluorochrome‐conjugated antibodies were obtained from eBioscience or Biolegend. Cells were stained and analysed by BD Calibur or BD LSRII using FlowJo software (Tree Star) as described 15. Cells were sorted with BD LSRII. Absolute cell counts were determined using CountBright™ Absolute Counting Beads (Invitrogen Inc, Carlsbad, CA, USA). The cell concentration (cells/μl) was calculated using the equation: cells/μl = number of cells events/number of beads events × assigned bead count of the lot/volume of sample (μl).

### Bone marrow differentiation *in vitro*

Bone marrow cells were prepared from the femurs of mice, and erythrocytes were depleted using ammonium chloride lysis buffer and then cocultured with CFSE‐labelled BaF3‐mock or BaF3‐RAE1ε cells at a ratio of 10:1 (bone marrow cells: BaF3 cells) in the presence of GM‐CSF (10 ng/ml) for 5 days. 10 μg/ml neutralizing anti‐NKG2D antibody (R&D Systems) was added into BaF3‐RAE1ε/bone marrow cells coculture system every 2 days as indicated. Resulting cells were stained and analysed using flow cytometry.

### Nitric oxide (NO) and arginase assay

Sorted MDSCs were plated at a density of 1.0 × 10^6^/ml per well for 24 hrs. The supernatants were collected for detecting cytokines and NO, and the cells were harvested for arginase assay. NO was measured using Griess reagents (Sigma‐Aldrich, St. Louis, MO, USA). Briefly, 100 μl supernatant was incubated with 100 μl Griess reagents for 10 min at 25°C and OD550 nm was measured. NO concentration was calculated according to the sodium nitrite standard curve. Arginase activity was detected in cell lysates using QuantiChrom™ Arginase Assay Kit from BioAssay Systems following the manufacturer's protocols.

### ELISA for cytokines

Mouse cytokines in sera or cell culture supernatants were measured using ELISA kits (Biolegend or R&D Systems) according to the manufacturer's protocols.

### MDSCs suppression assay

CD8^+^ T cells were labelled with CFSE and seeded into a 96‐well round‐bottom plate pre‐coated with 1 μg/ml anti‐CD3 (BD PharMingen) in the presence of 1 μg/ml soluble anti‐CD28 (BD PharMingen) antibodies. MDSCs were cocultured with CD8^+^ T cells at a 1:1 ratio. In some experiments, 10 μg/ml neutralizing anti‐IL‐10 (R&D Systems) or 500 μM nor‐NOHA (Cayman) was added to the culture medium. After 60 hrs, cells were stained for CD8 and CFSE dilution was analysed using flow cytometry.

### Adoptive transfer experiments

CT26 tumour cells (1.0 × 10^5^/mouse) were injected subcutaneously into BALB/c mice. 4T1 cells (1.0 × 10^5^/mouse) were injected into the mammary fat pads of female BALB/c mice. On days 3, 8 and 12 post‐tumour cell injections, 5.0 × 10^6^ MDSCs from BaF3‐mock or BaF3‐RAE1ε bearing mice were injected intravenously into tumour‐bearing mice. In some groups, mice treated with nor‐NOHA were injected subcutaneously for 10 consecutive days with nor‐NOHA (80 mg/kg) beginning on the same day they were injected with BaF3‐RAE1ε cells. In some groups, mice were injected with anti‐IL‐10 neutralizing antibody (250 μg/mouse) and BaF3‐RAE1ε cells simultaneously. Tumour size and survival were measured.

### Statistics

Data are shown as mean ± SD. To assess significance between two groups, data were analysed using Student's *t*‐test or Mann–Whitney test. To assess significance among multiple groups, data were analysed using one‐way ANOVA test. Survival curve was drawn by GraphPad Prism software, and survival analysis was performed by the log‐rank (Mantel–Cox) test.

## Results

### RAE‐1ε leads to the expansion of CD11b^+^Gr‐1^+^ myeloid cells *via* NKG2D

BaF3 cells are pro‐B cells and normally do not express any NKG2D ligands 16. To examine the role of RAE1ε on the expansion of CD11b^+^Gr‐1^+^ myeloid cells, BaF3 cells were transduced with a construct expressing RAE1ε gene (BaF3‐RAE1ε). As a control, BaF3 cells were transduced with an empty vector (BaF3‐mock). BaF3‐RAE1ε cells expressed high level of RAE1ε and could bind to NKG2D receptor as confirmed by flow cytometry (Fig. S1). To evaluate whether the transduction altered growth rate, BaF3‐mock and BaF3‐RAE1ε cells were plated at the same densities and counted every 24 hrs for 3 days. Both BaF3‐mock and BaF3‐RAE1ε cells had the same growth kinetics (data not shown), indicating that RAE1ε transduction did not affect the *in vitro* growth rate.

**Figure 1:**
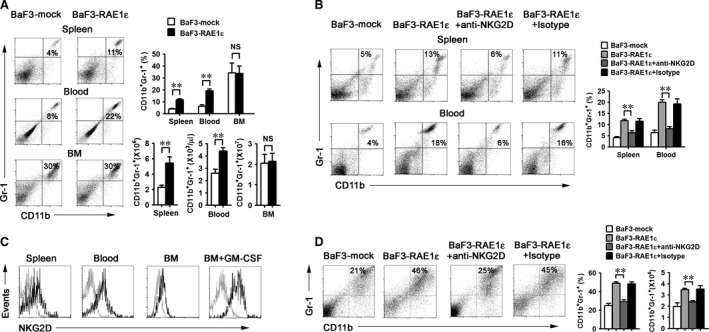
RAE1ε increases the expansion of CD11b^+^Gr‐1^+^ myeloid cells in peripheral tissues through NKG2D. (**A**) Mice injected with 4 × 10^6^ BaF3‐mock or BaF3‐RAE1ε tumour cells were killed at day 28 after injection. The frequencies of CD11b^+^Gr‐1^+^ cells in blood, spleen and bone marrow were measured by flow cytometry (*n* = 6 mice per group). Data are presented as mean ± SD of 3 mice. The absolute counts of CD11b^+^Gr‐1^+^ cells for blood (per μl), spleen and bone marrow (2 femurs and tibias) were calculated using CountBright™ Absolute Counting Beads. Data are presented as mean ± SD of 6 mice. (**B**) Mice injected with 4 × 10^6^ BaF3‐mock or BaF3‐RAE1ε tumour cells were killed at day 28 after injection. In some experiments, mice were injected with neutralizing anti‐NKG2D antibody (250 μg/mouse) or isotype‐matched control mAb (250 μg/mouse) through tail vein every 3 days. The frequencies of CD11b^+^Gr‐1^+^ cells in blood and spleen were measured by flow cytometry (*n* = 3–6 mice per group). Data are presented as mean ± SD of three mice. (**C**) Splenocytes, blood and bone marrow cells were harvested from tumour‐free mice. Some bone marrow cells were cultured with GM‐CSF for 5 days. These cells were triple labelled for CD11b, Gr1 and NKG2D. CD11b^+^Gr1^+^ cells were gated and analysed by flow cytometry for expression of NKG2D. Results are representative of three independent experiments. (**D**) 1.0 × 10^6^ bone marrow cells were cocultured with CFSE‐labelled 1.0 × 10^5^ BaF3‐mock or BaF3‐RAE1ε cells in the presence of GM‐CSF for 5 days. In some groups, neutralizing anti‐NKG2D antibody (10 μg/ml) was added into the BaF3‐RAE1ε/bone marrow cells coculture system every 2 days. Cells were harvested and stained with anti‐Gr‐1 and anti‐CD11b. The percentage and absolute number of CD11b^+^Gr‐1^+^ cells were determined in CFSE negative cells by flow cytometry. Results are representative of three independent experiments. Data are presented as mean ± SD of triplicate wells. **, *P* < 0.01; NS, not significant.

BaF3‐mock or BaF3‐RAE1ε cells were injected into mice, and the percentage and absolute number of CD11b^+^Gr‐1^+^ myeloid cells were measured. We found that the percent and absolute number of CD11b^+^Gr‐1^+^ myeloid cells were significantly elevated in spleen and blood of mice injected with BaF3‐RAE1ε (Fig. 1A). However, this difference was not detected in bone marrow of mice (Fig. 1A). NKG2D is the only known receptor for RAE1ε. To further confirm whether NKG2D mediated the effect of RAE1ε on CD11b^+^Gr‐1^+^ cells expansion, NKG2D‐blocking antibody was used to the mice injected with BaF3‐RAE1ε. As shown in Figure 1B, NKG2D blockade significantly reduced the CD11b^+^Gr‐1^+^ cell levels, indicating that RAE1ε mediates these cells accumulation through NKG2D *in vivo*. Moreover, no soluble RAE1ε was detected in BaF3‐RAE1ε cells culture supernatant (data not shown) and no difference in serum soluble RAE1ε production was observed among mice injected with BaF3‐mock or BaF3‐RAE1ε (Fig. S2), indicating that soluble RAE1ε was not involved in the expansion of the CD11b^+^Gr‐1^+^ myeloid cells.

Next, we assayed CD11b^+^Gr‐1^+^ cells from naive mice for NKG2D expression. As shown in Figure 1C, NKG2D was expressed on the surface of CD11b^+^Gr‐1^+^ cells in blood, spleen and bone marrow, with an increased expression upon the stimulation of GM‐CSF. Therefore, CD11b^+^Gr‐1^+^ cells express receptor for RAE1ε, making them potentially responsive to RAE1ε. To determine whether RAE1ε induces the differentiation of CD11b^+^Gr‐1^+^ cells, bone marrow cells were cultured with CFSE‐labelled BaF3‐mock or BaF3‐RAE1ε in the presence of GM‐CSF. NKG2D‐blocking antibody was added in the coculture system as indicated. After 5 days of culture, the percentage and the absolute number of CD11b^+^Gr1^+^ cells were determined in CFSE negative cells. As shown in Figure 1D, RAE1ε induced the CD11b^+^Gr‐1^+^ cell generation and blockade of NKG2D led to decrease in CD11b^+^Gr‐1^+^ cell levels. Taken together, these results demonstrated that RAE1ε mediates the generation of CD11b^+^Gr‐1^+^ cells *via* NKG2D.

### CD11b^+^Gr‐1^+^ MDSCs induced by RAE1ε have enhanced suppressive activity

**Figure 2:**
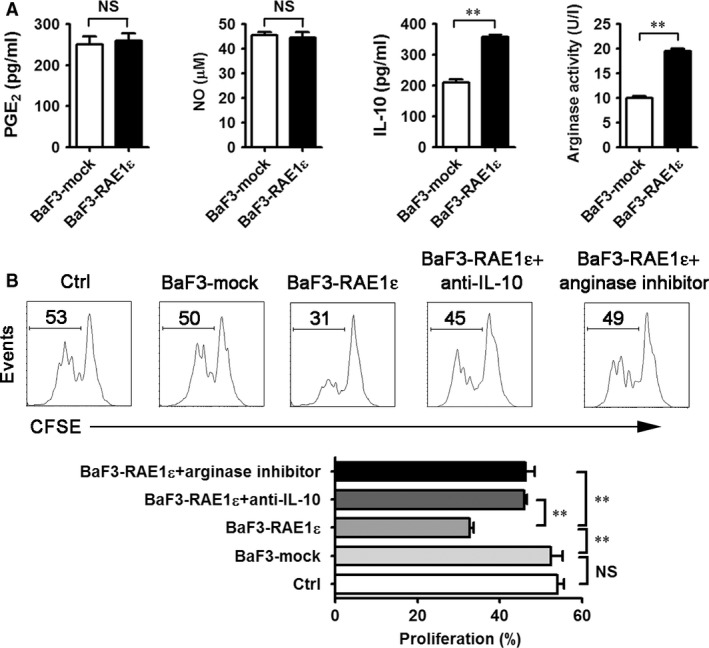
MDSCs from mice with BaF3‐RAE1ε cells have heightened suppressive activity. Mice were injected with BaF3‐mock or BaF3‐RAE1ε on day 0. On day 28, splenic CD11b^+^ cells were magnetically purified. (**A**) Aliquots of CD11b^+^ were cultured for 24 hrs. The supernatants were collected for determining PGE_2_, IL‐10 and NO production, and the cells were harvested for arginase assay, as described in Materials and methods. (**B**) Purified CD11b^+^ cells were cocultured with CFSE‐labelled, anti‐CD3‐/anti‐CD28‐activated CD8^+^ T cells. Arginase inhibitors (nor‐NOHA) or neutralizing anti‐IL‐10 were added to some wells. After 60 hrs, cell proliferation was determined as CFSE dilution by flow cytometry by gating on the CD8^+^ population. Representative histograms showing CFSE expression by the CD8^+^ T cells and the proliferation of CD8^+^ T cells was analysed. Results are representative of three independent experiments. Data are presented as mean ± SD of triplicate wells. **, *P* < 0.01; NS, not significant.

We wondered whether CD11b^+^Gr‐1^+^ myeloid cells induced by BaF3‐RAE1ε are phenotypically distinct from CD11b^+^Gr‐1^+^ myeloid cells induced by BaF3‐mock, mice were injected with BaF3‐mock or BaF3‐RAE1ε cells, and splenocytes were stained with CD11b, Gr‐1 and the indicated membrane molecules on day 28. CD40, CD80, B7H1, Tie2, CD206, CCR7, IL‐4R, IFN‐γR, IL‐10R, CD115, CD11c, F4/80 and dectin‐1 expressions were not significantly affected by RAE1ε (Fig. S3 and data not shown). Then, we wondered whether BaF3‐RAE1ε‐induced CD11b^+^Gr‐1^+^ cells have different functional activity from BaF3‐mock‐induced CD11b^+^Gr‐1^+^ cells, mice were injected with BaF3‐mock or BaF3‐RAE1ε cells, and splenic CD11b^+^ cells were sorted by MACS on day 28. Sorted cells were >90% CD11b^+^Gr‐1^+^ as measured by flow cytometry (data not shown). The functional properties of these sorted cells were detected. CD11b^+^ cells from BaF3‐RAE1ε‐bearing mice showed pronounced arginase activity and IL‐10 secretion, but failed to show increases in PGE_2_ and NO production (Fig. 2A). Moreover, no difference in IL‐4, IFN‐γ and TGF‐β production was detected among CD11b^+^ cells from BaF3‐mock or BaF3‐RAE1‐bearing mice (data not shown). Functionally, the ability of CD11b^+^ cells from BaF3‐RAE1ε‐bearing mice for inhibiting the proliferation of anti‐CD3‐/anti‐CD28‐stimulated CD8^+^ T cells significantly increased compared with that of CD11b^+^ cells from BaF3‐mock‐bearing mice (Fig. 2B). Thus, we conclude that RAE1ε induces splenic MDSCs with suppressive functions.

To determine whether IL‐10 and arginase could mediate the inhibitory effect of BaF3‐RAE1ε‐induced MDSCs on CD8^+^ T‐cell proliferation, we added nor‐NOHA (inhibitor of arginase) or anti‐IL‐10 antibody into the activated‐CD8^+^ T/MDSC coculture system and found that IL‐10 blockade or nor‐NOHA treatment could significantly reverse the suppression of the activated CD8^+^ T‐cell proliferation by BaF3‐RAE1ε‐induced MDSCs (Fig. 2B). Furthermore, we examined the role of BaF3‐RAE1ε MDSCs on Treg generation and found that RAE1ε did not promote generation of CD4^+^CD25^+^Foxp3^+^ Treg cells (Fig. S4). Together, these results have demonstrated that BaF3‐RAE1ε MDSCs are more effective suppressors of CD8^+^ T cells than are BaF3‐mock MDSCs. The suppressive function of BaF3‐RAE1ε MDSCs is arginase‐ and IL‐10‐dependent.

### RAE1ε affects the cytokine environment *in vivo*

**Figure 3:**
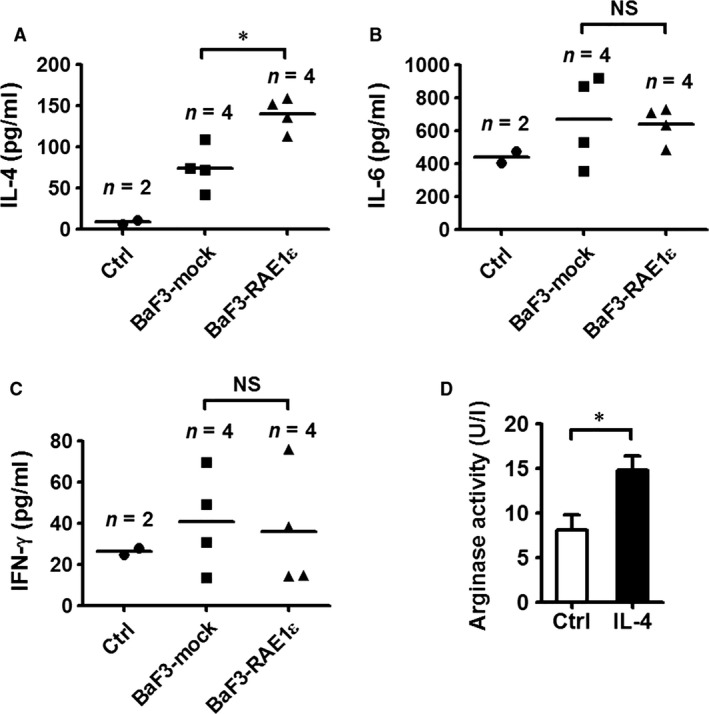
RAE1ε influences the cytokine environment *in vivo*. (**A–C**) Mice were injected with BaF3‐mock or BaF3‐RAE1ε on day 0. On day 28, sera were collected for the measurement of IL‐4 (**A**), IL‐6 (**B**) and IFN‐γ (**C**) levels by ELISA. Tumour‐free mice were used as a control. (**D**) Mice were injected with BaF3‐mock on day 0. On day 28, splenic CD11b^+^ cells were magnetically purified. Purified CD11b^+^ cells were stimulated with 100 ng/ml recombinant murine IL‐4 for 24 hrs and then harvested for arginase assay. Results are representative of three independent experiments. Data are presented as mean ± SD of triplicate wells. *, *P* < 0.05; NS, not significant.

Different types of cytokines have been implicated in driving MDSC expansion and supporting MDSC immunosuppressive activity 17, 18, 19, 20. Therefore, several related cytokines were measured in the sera of mice injected with BaF3‐mock or BaF3‐RAE1ε. As shown in Figure 3A, IL‐4 was increased in the sera of mice injected with BaF3‐RAE1ε cells compared with the BaF3‐mock cells. No significant differences in IL‐6 (Fig. 3B), IFN‐γ (Fig. 3C) and TGF‐β (data not shown) production in the sera of mice injected with BaF3‐mock or BaF3‐RAE1ε cells were observed. IL‐12p70 and TNF‐α were not detected in either group of mice (data not shown). Previous studies have shown that IFN‐γ induced NO production and IL‐4 induced arginase production from MDSCs 21, 22, 23. We found that CD11b^+^ cells from BaF3‐mock‐bearing mice exhibited enhanced arginase activity upon IL‐4 stimulation (Fig. 3D). So, the increased IL‐4 is potentially involved in the pronounced arginase activity in MDSCs from BaF3‐RAE1ε‐injected mice in Figure 2A.

### RAE1ε‐induced MDSCs promote tumour growth

**Figure 4:**
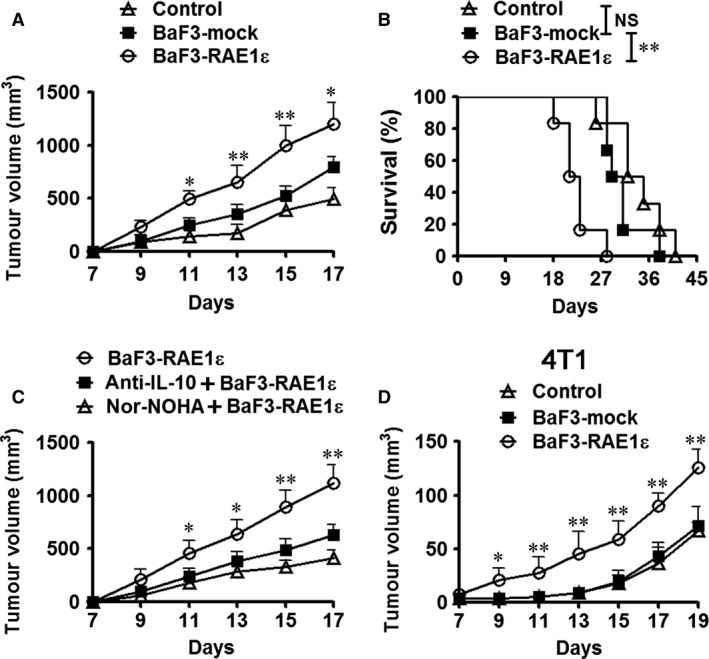
MDSCs from BaF3‐RAE1ε‐injected mice promote tumour growth. (**A**,** B**) CT26 tumour cells were injected subcutaneously into BALB/c mice. MDSCs from BaF3‐mock or BaF3‐RAE1ε bearing mice were adoptively transferred by tail vein injection on days 3, 8 and 12 after CT26 tumour cell injection. Tumour volume and survival rates were monitored. Each group included six mice. (**C**) CT26 tumour cells were injected subcutaneously into BALB/c mice. MDSCs from BaF3‐RAE1ε bearing mice were adoptively transferred by tail vein injection on days 3, 8 and 12 after CT26 tumour cell injection. In some groups, anti‐IL‐10 neutralizing antibody and BaF3‐RAE1ε cells administration simultaneously. In some groups, mice were injected for 10 consecutive days with nor‐NOHA beginning on the same day they were injected with BaF3‐RAE1ε. Tumour volume was monitored. (**D**) 4T1 tumour cells were injected into the mammary fat pads of female BALB/c mice. MDSCs from BaF3‐mock or BaF3‐RAE1ε bearing mice were adoptively transferred by tail vein injection on days 3, 8 and 12 after tumour inoculation. Tumour volume was monitored. Each group included seven mice. *, *P* < 0.05; **, *P* < 0.01; NS, not significant.

MDSCs have been reported to support tumour progression by suppressing antitumour immune responses 11. MDSCs induced by RAE1ε were identified to have enhanced suppressive activity in Figure 2A‐B. Therefore, we analysed the effect of BaF3‐RAE1ε MDSCs on tumour growth *in vivo*. Mice were subcutaneously transplanted with CT26 tumour cells and then adoptive transfer of BaF3‐mock MDSCs or BaF3‐RAE1ε MDSCs. We found that tumour growth and survival rates were not significantly affected after adoptive transfer of BaF3‐mock MDSCs (Fig. 4A, B). However, BaF3‐RAE1ε MDSCs adoption significantly promoted subcutaneous tumour growth (Fig. 4A) and reduced survival time (Fig. 4B). To further confirm that arginase and IL‐10 are involved in the pro‐tumour function of BaF3‐RAE1ε MDSCs, CT26‐bearing mice adoptively transferred with BaF3‐RAE1ε MDSCs were treated with anti‐IL‐10 antibody or nor‐NOHA. As shown in Figure 4C, a delay in tumour growth was observed as long as anti‐IL‐10 or nor‐NOHA was administered, suggesting that BaF3‐RAE1ε MDSCs promote tumour growth in IL‐10‐ and arginase‐dependent manners. In addition, we found that BaF3‐RAE1ε MDSCs could also enhance 4T1 mammary carcinomas growth *in vivo* (Fig. 4D).

### RAE1ε enhances suppressive activity of MDSCs through IL‐10 *in vitro*

**Figure 5:**
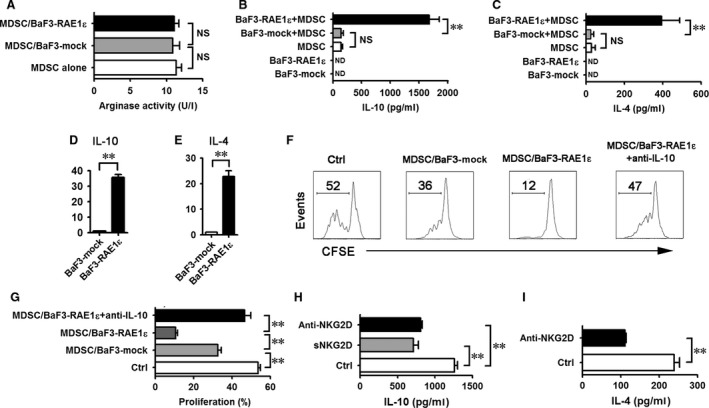
MDSCs treated with BaF3‐RAE1ε produce increased levels of IL‐4 and IL‐10 *in vitro*. (**A**–**E**) Mice were injected with CT26 tumour cells on day 0. On day 28, splenic MDSCs were magnetically purified and then cocultured with BaF3‐mock or BaF3‐RAE1ε cells at a ratio of 10:1 (MDSC: BaF3) for 24 hrs. (**A**) MDSCs were sorted from the cocultured system for detecting arginase activity. (**B**,** C**) The supernatants were collected for determining IL‐10 and IL‐4 production by ELISA. (**D**,** E**) MDSCs were sorted from the cocultured system for checking IL‐10 and IL‐4 production by quantitative RT‐PCR. IL‐10 or IL‐4 expression in MDSCs post‐cocultured with BaF3‐mock was designated as 1. (**F**,** G**) MDSCs were sorted from the cocultured system and then cocultured with CFSE‐labelled, anti‐CD3‐/anti‐CD28‐activated CD8^+^ T cells. Neutralizing anti‐IL‐10 was added to some wells. After 60 hrs, cell proliferation was determined as CFSE dilution by flow cytometry by gating on the CD8^+^ population. Representative histograms showing CFSE expression by the CD8^+^ T cells (**F**) and the proliferation of CD8^+^ T cells was analysed (**G**). (**H**,** I**) MDSCs were pre‐treated with 10 μg/ml soluble NKG2D for 1 hr and then cocultured with BaF3‐RAE1ε, or BaF3‐RAE1ε were pre‐treated with 10 μg/ml anti‐NKG2D for 1 hr and then cocultured with MDSCs. After 24 hrs, the level of IL‐10 and IL‐4 in supernatants was determined by ELISA. MDSCs cocultured with BaF3‐RAE1ε were used as a positive control. Results are representative of three independent experiments. Data (except **F**) are presented as mean ± SD of triplicate wells. **, *P* < 0.01; NS, not significant.

Because NKG2D was identified to be expressed on CD11b^+^Gr‐1^+^ cells in Figure 1C, we sought to determine whether RAE1ε is directly enhancing the immunosuppressive function of MDSCs *in vitro*. Highly purified MDSCs from CT26 cell‐injected mice were cocultured with BaF3‐mock or BaF3‐RAE1ε for 24 hrs, and then, the MDSCs were sorted for testing the immunosuppressive function. As shown in Figure 5A, BaF3‐RAE1ε did not affect arginase production of MDSCs. It contrasts to the results that show that MDSCs from BaF3‐RAE1ε‐bearing mice exhibited pronounced arginase activity (Fig. 2A), suggesting that RAE1ε may indirectly activate MDSCs to produce arginase *in vivo*. Furthermore, we found that MDSCs activated by BaF3‐RAE1ε secreted higher level of IL‐10 and IL‐4 (Fig. 5B‐E). There was no obvious difference in secretion of IFN‐γ, IL‐6, PGE_2,_ TGF‐β and NO by MDSCs cocultured with or without BaF3‐RAE1ε (data not shown). MDSCs activated by BaF3‐RAE1ε significantly inhibited CD8^+^ T‐cell proliferation *via* IL‐10 (Fig. 5F, G), which resembled the functional characteristics of MDSCs induced by BaF3‐RAE1ε *in vivo*. To further determine whether RAE1ε‐NKG2D pathway is involved in the high IL‐4 and IL‐10 production of MDSCs, soluble NKG2D or neutralizing anti‐NKG2D antibody was added into the BaF3‐RAE1ε/MDSC coculture system, and then, the IL‐4 and IL‐10 secretion in supernatants was measured. As expected, blockade of RAE1ε‐NKG2D interaction impaired IL‐4 and IL‐10 production (Fig. 5H, I), indicating that BaF3‐RAE1ε induced IL‐10 and IL‐4 production in MDSCs *via* NKG2D.

## Discussion

It has been well‐established that NKG2D ligands expressed by tumour cells activate T cells and NK cells *via* NKG2D, and thus playing important roles in favouring tumour surveillance 2. However, tumour infiltrating and systemic cytolytic cells usually express low level of NKG2D and show dysregulation 24. Numerous potential mechanisms have been proposed. For example, engagement of soluble NKG2D ligands cleaved from solid tumours promotes NKG2D internalization and degradation, and thus compromising the NKG2D‐dependent immune cell activation 25. In agree with this, soluble NKG2D ligands have been shown to be associated with tumour progression and poor prognosis in several types of tumours 4, 26, 27. In addition, sustained NKG2D ligand expressed by tumours also down‐regulates NKG2D, which impairs cytotoxicity of immune cells and promotes tumour escape 3. However, no difference in the expression of NKG2D by NK cells and CD8^+^ T cells was observed among mice injected with BaF3‐mock or BaF3‐RAE1ε in our system (data not shown). Tumours have developed complex mechanisms to dampen immune responses, such as employing immunosuppressive cells 20, 28. We have been suggested that NKG2D ligands^+^ tumour cells may also have evolved strategy to negatively regulate immune responses by generation of the immunosuppressive cells. To test our hypothesis, we applied our previously constructed RAE1ε expressing BaF3 cells and BaF3‐mock control cells on mice and compared their ability to induce immunosuppressive cells. We found that RAE1ε did not promote generation of CD4^+^CD25^+^Foxp3^+^ Treg cells (Fig. S4B), CD19^+^Tim‐1^+^ or CD19^+^CD1d^hi^CD5^+^ regulatory B cells in spleen (data not shown). Interestingly, RAE1ε affected MDSCs in two ways: inducing their generation and increased their suppressive function, which may represent a novel mechanism used by tumour cells to escape from immunosurveillance.

Recently, soluble NKG2D ligand MIC promoting accumulation of MDSCs *via* NKG2D has been reported 29. No soluble RAE1ε was detected in BaF3‐RAE1ε cells culture supernatant, and soluble RAE1ε levels in sera were similar for BaF3‐mock and BaF3‐RAE1ε‐injected mice, confirming that the GPI‐anchored RAE‐1ε was not cleaved by proteolysis 15, 16. These data suggested that it is surface NKG2D ligand RAE1ε, rather than soluble RAE1ε, that induces the accumulation of MDSCs in our experiment system. Moreover, surface RAE1ε was directly involved in the accumulation of MDSCs as administration of the anti‐NKG2D antibody inhibited the accumulation of this cell population. Our current results and other previous studies have demonstrated that NKG2D ligands, both surface and soluble types, play important roles in driving generation of MDSCs. Furthermore, our experiments did not capture the observed difference in phenotype when comparing MDSCs from BaF3‐mock‐ and BaF3‐RAE1ε‐injected mice, indicating that RAE1ε may be accumulating the existing population of MDSCs, but not inducing a new population.

Functionally, suppression of T cells is an important characteristic of MDSCs. There are at least two kinds of mechanisms that may account for regulatory function of MDSCs, including suppressing antigen‐specific/antigen‐non‐specific T‐cell proliferation or inducing Treg cell generation 21. While the proliferation of CD8^+^ T cell was indeed slightly inhibited by CD11b^+^Gr‐1^+^ cells from BaF3‐mock‐injected mice, this did not reach statistical significance (Fig. 2B, C). In contrast, the proliferation of CD8^+^ T cells was significantly suppressed by MDSCs from CT26 tumour‐bearing mice (Fig. 5F, G). These findings are in agreement with previous reports that the variability of MDSCs efficiency to inhibit T‐cell responses depends on the type and stage of tumour 30. A significantly increased production of IL‐10 and activity of arginase was observed in MDSCs purified from BaF3‐RAE1ε‐bearing mice compared to BaF3‐mock‐bearing mice, which was accompanied by enhanced suppressive activity as indicated by inhibiting T‐cell proliferation in response to anti‐CD3/anti‐CD28. However, increased IL‐10 production was significantly observed in CT26 tumour‐derived MDSCs activated by BaF3‐RAE1ε *in vitro*, and arginase activity remained unchanged. There are two possibilities: (i) different tumour‐derived MDSCs exhibit different effect in response to RAE1ε; (ii) RAE1ε induces arginase production by MDSCs indirectly. We will address these questions using more different tumour‐derived MDSCs in future investigations.

Tumour‐derived mediators, such as TGF‐β, IL‐1β and IL‐6, as well as cytokines produced by T cells, such as IFN‐γ, IL‐4 and IL‐10, promote MDSCs population expansion and support their immunosuppressive activity 31. For example, MDSCs inhibit T‐cell function through production of NO and arginase, which are dependent on IFN‐γ and IL‐4 or IL‐13 stimulation, respectively 21, 32, 33, 34. We found that RAE1ε did not affect IL‐6, IFN‐γ, TGF‐β, IL‐12p70 or TNF‐α production in sera of mice (data not shown). However, the level of IL‐4 was increased when RAE1ε was present compared with controls, which may confer an enhanced arginase activity in MDSCs from BaF3‐RAE1ε‐injected mice. Our previous studies show that BaF3‐RAE1ε could induce NK cells and CD8 T cells to release high level of IFN‐γ *in vitro* 15, 35. Interestingly, BaF3‐RAE1ε induced CT26‐derived MDSCs to release IL‐4 rather than IFN‐γ in this study. IFN‐γ belongs to Th1 type cytokines while IL‐4 belongs to Th2‐type cytokines, and they play opposite roles in immune responses 22, 36. Our results suggested that upon RAE1ε binding to NKG2D, the net outcome of activation or inhibition of immune response is determined by the cell type which expressed NKG2D.

In conclusion, we demonstrate that surface RAE1ε could promote accumulation of CD11b^+^Gr‐1^+^ MDSCs with high IL‐10 and arginase production. These MDSCs regulate immune response by inhibiting CD8 T‐cell proliferation. RAE1ε induces MDSCs to produce IL‐4 *via* NKG2D *in vitro*. Therefore, tumour surface NKG2D ligands may have evolved strategies to negatively regulate immune responses by facilitating the proliferation and the inhibitory functions of MDSCs.

## Conflict of interest

The authors declare no competing financial interests.

## Supporting information


**Figure S1** BaF3‐RAE1ε cells express high level RAE1ε.
**Figure S2** Soluble RAE1ε concentrations in sera are similar for mice injected with BaF3‐mock and BaF3‐RAE1ε.
**Figure S3** CD11b^+^Gr‐1^+^ cells from mice with BaF3‐mock and CD11b^+^Gr‐1^+^ cells from mice with BaF3‐RAE1ε have no phenotypic differences.
**Figure S4** MDSC from mice with BaF3‐RAE1ε and MDSC from mice with BaF3‐mock have no difference in Treg cell induction.
