# Supplementary material for: NKG2D ligand RAE1ε induces generation and enhances the inhibitor function of myeloid‐derived suppressor cells in mice
Source: J Cell Mol Med. 2017 Mar 9;21(9):2046–54. doi: 10.1111/jcmm.13124 (PMC5571551; doi:10.1111/jcmm.13124)
Supplement: Supplementary file 1 — Figure S1 BaF3‐RAE1ε cells express high level RAE1ε. Figure S2 Soluble RAE1ε concentrations in sera are similar for mice injected with BaF3‐mock and BaF3‐RAE1ε. Figure S3 CD11b+Gr‐1+ cells from mice with BaF3‐mock and CD11b+Gr‐1+ cells from mice with BaF3‐RAE1ε have no phenotypic differences. Figure S4 MDSC from mice with BaF3‐RAE1ε and MDSC from mice with BaF3‐mock have no difference in Treg cell induction. [file JCMM-21-2046-s001.docx]

**Supplemental materials**

**Figure S1. BaF3-RAE1ε cells express high level RAE1ε.** (A) BaF3-mock or BaF3-RAE1ε cells were stained with rat anti-mouse RAE1ε Ab followed by Alexa Fluor® 488 conjugated donkey anti-rat IgG secondary Ab. (B) BaF3-mock or BaF3-RAE1ε cells were stained with recombinant mouse NKG2D/human IgG Fc chimera and followed by FITC conjugated goat anti-human IgG secondary Ab. Results are representative of at least three independent experiments.


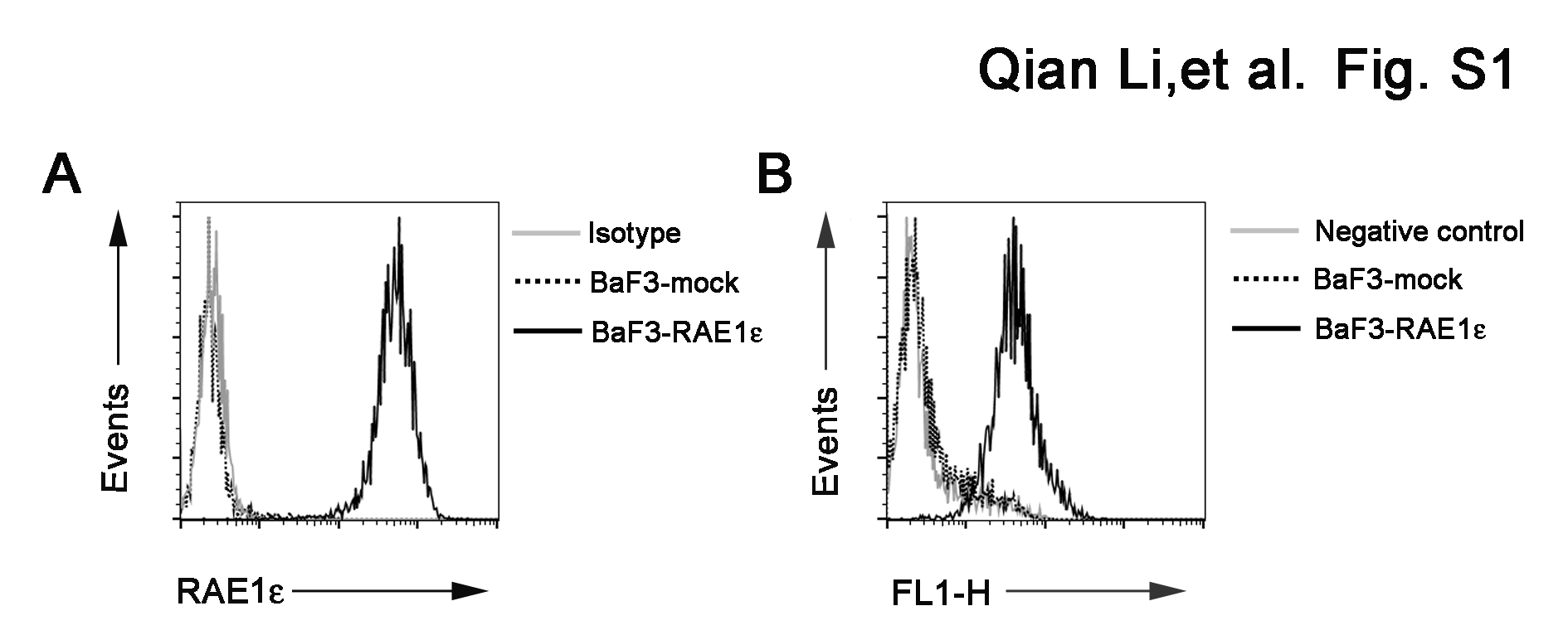


**Figure S2. Soluble RAE1ε concentrations in sera are similar for mice injected with BaF3-mock and BaF3-RAE1ε.** Mice injected with BaF3-mock or BaF3-RAE1ε cells were killed at day 28 after injection. Serum soluble RAE-1ε concentration was determined with a sandwich ELISA kit from R&D Systems (n=4 mice per group). Data were shown as mean ± SD. NS indicates not significant.


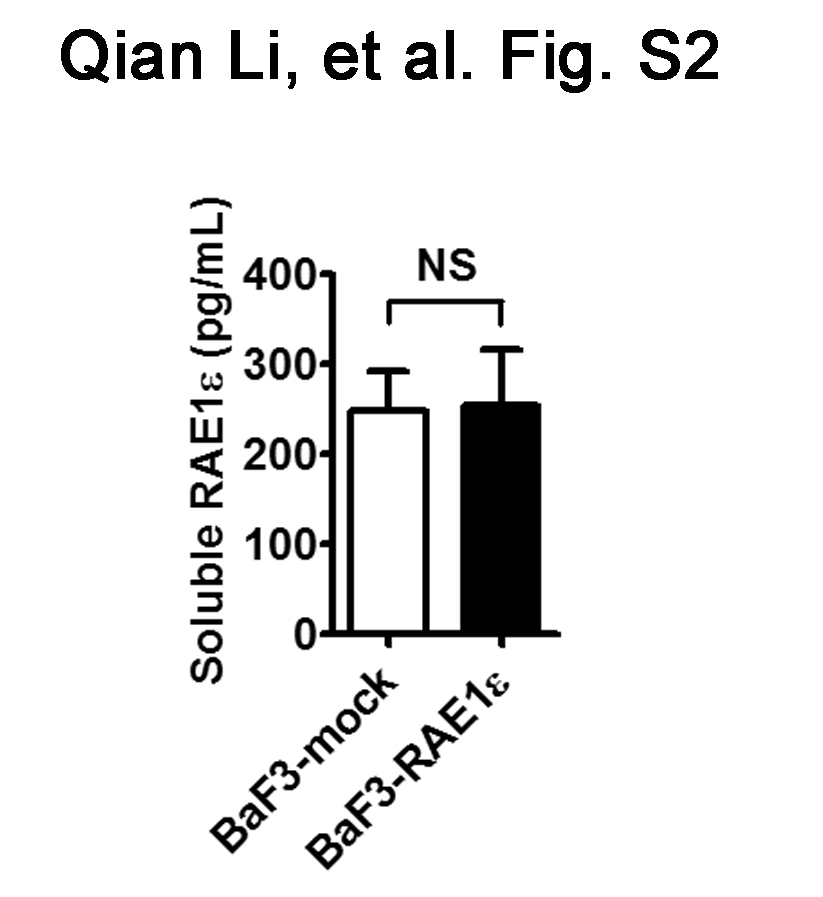


**Figure S3. CD11b^+^Gr-1^+^ cells from mice with BaF3-mock and CD11b^+^Gr-1^+^ cells from mice with BaF3-RAE1ε have no phenotypic differences.** Mice injected with 4×10^6^ BaF3-mock or BaF3-RAE1ε tumor cells were killed at day 28 after injection. Expression of CD40, CD80, B7H1, Tie2, CD206, CCR7, IL-4R and IFN-γ was evaluated in the splenic CD11b^+^Gr-1^+^ cells. Results are representative of three independent experiments.

**
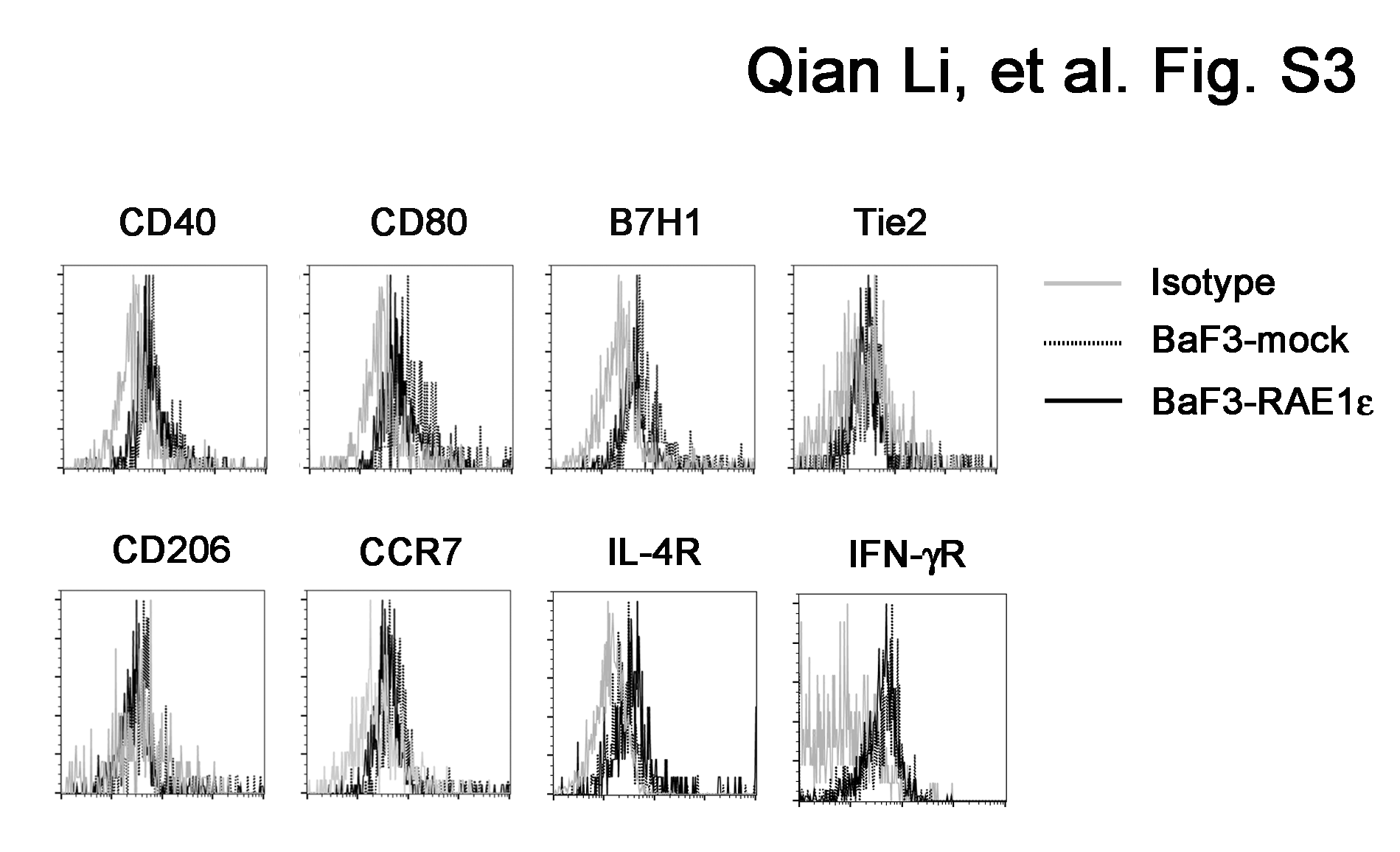
**

**Figure S4. MDSC from mice with BaF3-RAE****1ε and MDSC from mice with BaF3-mock have no difference in Treg cell induction.** (A) Splenic MDSCs from BaF3-mock or BaF3-RAE1ε injected mice were cocultured with anti-CD3/anti-CD28 activated CD4^+^ T cells at a 1:1 ratio for 5 days and subsequently stained with CD4, CD25, and Foxp3 antibodies. The CD4^+^ cell population was gated to show Treg cell percentages. (B) Mice injected with BaF3-mock or BaF3-RAE1ε cells were killed at day 28 after injection. The percent of CD4^+^CD25^+^foxp3^+^ Treg in splenocytes was measured by flow cytometry. Results are representative of three independent experiments.

**
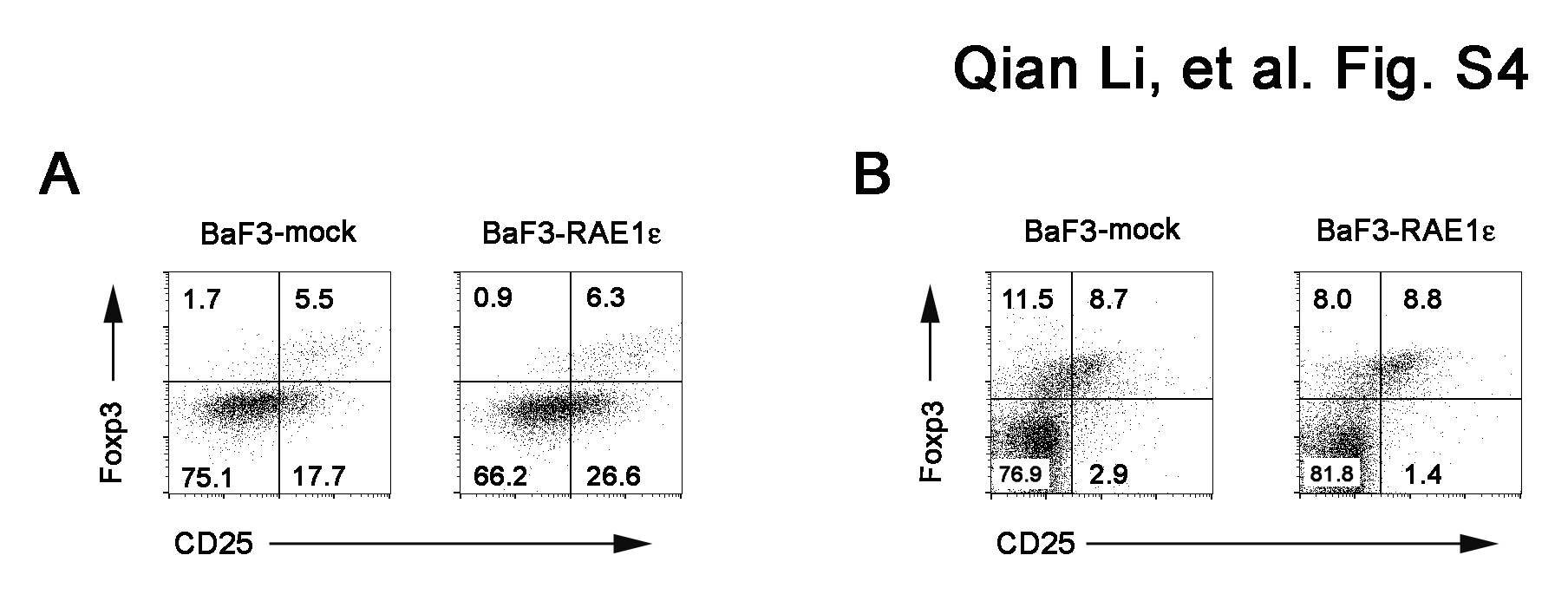
**
